# Lexico-semantic and acoustic-phonetic processes in the perception of noise-vocoded speech: implications for cochlear implantation

**DOI:** 10.3389/fnsys.2014.00018

**Published:** 2014-02-25

**Authors:** Carolyn McGettigan, Stuart Rosen, Sophie K. Scott

**Affiliations:** ^1^Department of Psychology, Royal Holloway, University of LondonEgham, UK; ^2^Institute of Cognitive Neuroscience, University College LondonLondon, UK; ^3^Department of Speech, Hearing and Phonetic Sciences, University College LondonLondon, UK

**Keywords:** speech perception, individual differences, cochlear implants

## Abstract

Noise-vocoding is a transformation which, when applied to speech, severely reduces spectral resolution and eliminates periodicity, yielding a stimulus that sounds “like a harsh whisper” (Scott et al., 2000, p. 2401). This process simulates a cochlear implant, where the activity of many thousand hair cells in the inner ear is replaced by direct stimulation of the auditory nerve by a small number of tonotopically-arranged electrodes. Although a cochlear implant offers a powerful means of restoring some degree of hearing to profoundly deaf individuals, the outcomes for spoken communication are highly variable (Moore and Shannon, 2009). Some variability may arise from differences in peripheral representation (e.g., the degree of residual nerve survival) but some may reflect differences in higher-order linguistic processing. In order to explore this possibility, we used noise-vocoding to explore speech recognition and perceptual learning in normal-hearing listeners tested across several levels of the linguistic hierarchy: segments (consonants and vowels), single words, and sentences. Listeners improved significantly on all tasks across two test sessions. In the first session, individual differences analyses revealed two independently varying sources of variability: one lexico-semantic in nature and implicating the recognition of words and sentences, and the other an acoustic-phonetic factor associated with words and segments. However, consequent to learning, by the second session there was a more uniform covariance pattern concerning all stimulus types. A further analysis of phonetic feature recognition allowed greater insight into learning-related changes in perception and showed that, surprisingly, participants did not make full use of cues that were preserved in the stimuli (e.g., vowel duration). We discuss these findings in relation cochlear implantation, and suggest auditory training strategies to maximize speech recognition performance in the absence of typical cues.

## Introduction

A cochlear implant (CI) is a hearing aid that converts acoustic sound energy into electrical stimuli to be transmitted to the auditory nerve, via an array of electrodes arranged tonotopically along the basilar membrane of the inner ear (Rubinstein, 2004). Although the implant restores some degree of hearing to profoundly deaf individuals, the substitution of thousands of inner hair cells with, at most, tens of electrodes means that the transmitted signal is greatly impoverished in spectral detail. CI devices give a weak sense of voice pitch and transmit fewer discriminable steps in amplitude, and there is often a mis-match between frequencies being transmitted by the individual electrodes and those best received at the position of contact on the basilar membrane. Thus, particularly for post-lingual recipients of an implant (i.e., those who lost their hearing after the acquizition of language), the listener must learn to make sense of an altered and unfamiliar sound world. This process of adaptation and perceptual learning after cochlear implantation can take a long time, with widely varying levels of success (Pisoni, 2000; Sarant et al., 2001; Munson et al., 2003; Skinner, 2003). Much research relating to implantation has therefore been concerned with identifying predictive markers of success, and appropriate training regimes to optimize post-implantation outcomes.

A growing body of studies has employed acoustic simulations of CIs to model post-implantation adaptation in normal-hearing participants. Vocoding is an acoustic transformation that produces speech with degraded spectral detail by replacing the original wideband speech signal with a variable number of amplitude-modulated carriers, such as noise bands (noise-vocoding) or sine waves (tone vocoding). Here, the carriers simulate the electrodes of the CI to create a re-synthesized speech signal that is spectrally impoverished, yet maintains relatively intact amplitude envelope cues (Shannon et al., 1995). Here, increasing the number of bands (or channels) increases the spectral resolution, with a concomitant increase in intelligibility of transformed sounds. As with the studies in CI recipients, it has been shown that normal-hearing participants also exhibit considerable individual variability in performance with CI simulations (Nogaki et al., 2007; Stacey and Summerfield, 2007; Eisner et al., 2010).

An important issue for clinicians testing and training CI recipients is the selection of appropriate materials (Loebach and Pisoni, 2008; Loebach et al., 2008, 2009). The main day-to-day context for spoken communication is spontaneous face-to-face conversation, so it is natural to consider training paradigms similar to that situation. For example, connected discourse tracking (CDT), using face-to-face repetition of a story told by an experimenter, has been shown to yield significant improvements in the recognition of severely degraded speech in hearing participants (Rosen et al., 1999). However, delivery of this kind of training is very labor-intensive (though a recent study comparing live CDT with a computer-based approach showed equivalent training benefits from the two training routines; Faulkner et al., 2012). Thus, training and assessment routines typically involve the recognition of laboratory recordings of materials such as sentences, words and simple syllables. There is some evidence that improvements with one kind of test material can generalize to another. For example, Loebach and Pisoni (2008) found that training participants with exposure and feedback on either words, sentences or environmental sounds gave improvements in performance that generalized to the other tasks. In a very small group of three CI recipients, Fu et al. (2005) found that training with CV and CVC syllables (where “C” stands for Consonant, and “V” for Vowel) led to improved test performance on sentence recognition. However, the picture is not straightforward: Loebach and Pisoni (2008) found that generalization was most effective between materials of the same class (e.g., words to words, sentences to sentences), and that training on speech materials did not afford any improvements in recognition of environmental sounds. This may depend on the nature of the vocoding transformation, as this severely degrades spectral detail important for recognizing some environmental sounds—similarly, training on vocoded sentence materials gave poor generalization to the recognition of talkers (Loebach et al., 2009).

Imbalances in learning transfer may also be affected by the behavior of the listener. Loebach et al. (2008) found that training on talker identification afforded greater generalization to vocoded sentence transcription than training on talker gender identification. The authors suggest this is because the more difficult task of talker identification led to greater attentional engagement of the listeners with the acoustic properties of vocoded speech. Similarly, Loebach et al. (2010) and Davis et al. (2005) found that training participants with semantically anomalous sentences was just as effective as training with meaningful sentences, while Hervais-Adelman et al. (2008) found equivalent improvements in performance after training with vocoded non-words as observed with real-word training. Loebach et al. (2010) suggest this is because the absence of semantic cues engages a more analytic listening mode in the listener, where attention is directed to the acoustic-phonetic aspects of the signal rather than “synthetic,” higher-order processes focused on linguistic comprehension. Although they acknowledge that sentence and word materials afford greater ecological validity (Loebach et al., 2010), Loebach and colleagues suggest that encouraging analytic, acoustic-phonetic, listening can afford better generalization of learning across a range of materials.

Another way to view the data from these training studies is that there are potentially both “analytic” and “synthetic” factors at play when listeners adapt to degraded speech input. However, the extent to which these operate independently is not known, and it may be that some listeners would stand to benefit more from training on higher-order processes for employment in the recognition of ecologically valid, linguistic materials encountered in day-to-day life. A study by Grant and Seitz (2000) showed that use of “top–down” contextual information—what Loebach and colleagues would consider a “synthetic” process—varies across individuals. They presented 34 hearing-impaired listeners with filtered sentences from the IEEE corpus (e.g., “Glue the sheet to the dark blue background”; IEEE, 1969), and their constituent keywords in isolation, at three different intelligibility levels. Using Boothroyd and Nittrouer's (1988) equation explaining the relationship between word recognition in sentences and in isolation, Grant and Seitz (2000) calculated individual k-factor scores—representing the listener's ability to use semantic and morpho-syntactic information in the sentence to identify the words within it—and observed considerable variability in this parameter across their listening population. In further support of multiple factors underlying speech recognition, Surprenant and Watson's (2001) large-scale study of individual variability in speech-in-noise recognition indicated that performance is far from identical across different linguistic levels—Pearson's correlation coefficients between speech-in-noise recognition of CV-units, words and sentences and a clear-speech syllable identification task ranged from only 0.25 to 0.47 in their experiment. Therefore, extracting patterns of covariance, and measuring how these change with learning, could offer additional insight into the underlying perceptual processes supporting adaptation to a CI or simulation. For example, close correlation of speech recognition at segment, word and sentence level may indicate a unified “analytic” strategy, whereas statistical independence of sentence stimuli from words and segments could reflect considerable importance for top–down syntactic and semantic processing strategies in recognizing vocoded sentences.

In the current experiment, we tested a group of normal-hearing adults in the transcription of noise-vocoded sentences, words and segments at a range of difficulty levels (operationalized in terms of the number of vocoding bands), with the following objectives:

- *To explore patterns of covariation across the linguistic hierarchy*—segment, word, sentence—in order to characterize the number and nature of factors underlying the recognition of vocoded speech. We predicted that individual scores across the five tasks would be significantly correlated, but that individuals' differing abilities to use “top–down”/“synthetic” and “bottom–up”/“analytic” processes would limit the strength of these correlations.
- *To measure long-term perceptual learning of vocoded speech*—by re-testing participants after 1–2 weeks. There were several aims here:
  - To assess whether adaptation to noise-vocoded speech can be maintained over several days without exposure.
  - To compare the size of any adaptation effects across stimulus type.
  - To explore whether adaptation / additional exposure alters listening strategies (as measured using analyses of covariation).
  Davis et al. (2005) found that transcription of vocoded sentences improved significantly within 30 items of exposure, in the absence of feedback. Therefore, to minimize design complexity and optimize the exploitation of individual differences analyses, learning in the current experiment was operationalized as the improvement in performance from Session 1 to Session 2, without involvement of explicit training procedures.
- *To quantify the efficiency of “analytic” listening*—Loebach et al. (2010) suggested that failure to attend to critical acoustic properties of vocoded stimuli may limit the transfer of learning. Our final objective was to use information transfer (IT) analyses of consonant and vowel perception to quantify the reception of acoustic-phonetic features, and to directly assess the degree to which the acoustic cues present in the stimulus are being used by untrained listeners.

Previous work on the learning of vocoded speech has tended to train and test participants at a fixed level of degradation (i.e., number of bands; Davis et al., 2005; Hervais-Adelman et al., 2008; Loebach and Pisoni, 2008; Eisner et al., 2010). Given the considerable variation in individual performance with vocoded stimuli, this runs the risk of floor or ceiling effects in the data. For the current experiment, we adopted an approach used by Shannon et al. (2004), who tested across a range of difficulty levels (numbers of channels) and fitted logistic functions to describe performance on a range of noise-vocoded speech recognition tasks. In the current experiment, curves were fitted to the recognition data for each participant, by task and by session—estimates of 50% thresholds, representing performance across a range of difficulty, could then be extracted for use in further analysis of learning effects and covariation across tasks and time.

## Method

### Participants

Participants were 28 monolingual speakers of British English (12 male), with no language or hearing problems. The participants were recruited from the UCL Department of Psychology Subject Pool using an age inclusion criterion of 18–40 years old (individual date of birth information was not collected). All participants were naïve to noise-vocoded speech.

### Materials

Listeners were tested on perception of 5 different stimulus types, all vocoded with 1, 2, 4, 8, 16, and 32 bands (where 1 is most degraded, and 32 the most intelligible). The items were also available in undistorted form. All materials were recorded by a female speaker of Standard Southern British English in a soundproof, anechoic chamber. Recordings were made on a Digital Audio Tape recorder (Sony 60ES) and fed to the S/PDIF digital input of an M-Audio Delta 66 PC soundcard. The files were then downsampled at a rate of 44100 Hz to mono.wav files with 16-bit resolution using Cool Edit 96 software (Syntrillium Software Corporation, USA). The vocoding algorithm followed the general scheme described by Shannon et al. (1995), with analysis and output filters between 100–5000 Hz and envelope extraction via half-wave rectification and low-pass filtering at 400 Hz.

The stimulus sets were as follows:

- **Simple Sentences.** One-hundred-and-forty items from the BKB sentence corpus (Bench et al., 1979), each with three keywords (e.g., The *clown* had a *funny face*).
- **Low Predictability Sentences.** One-hundred-and—forty items from the IEEE sentence corpus (IEEE, 1969), each with five keywords (e.g., The *birch canoe slid* on the *smooth planks*).
- **Single Words.** One-hundred-and-forty items from the phonemically-balanced Boothroyd AB lists (e.g., gas, mice, whip; Boothroyd, 1968).
- **Consonants.** Seventeen consonants: b, d, f, g, dȝ, k, l, m, n, p, s, ʃ, t, v, w, j, z. One token of each consonant was recorded in the context /ɑ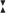/-C-/ɑ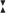/, where C is a consonant e.g., *apa, aga, ala*.
- **Vowels.** Seventeen vowels: æ, eɪ, ɑ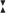, ε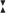, i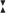, iə, e, ɪ, aɪ, ɜ, ɒ, əʊ, u:, ɔ, aʊ, ɔʊ, Λ. One token of each vowel was recorded in the context /*b*/-*V*-/*d*/, where V is the vowel e.g., *bad*, *beard*, *boyed*.

### Design and procedure

Twenty-seven listeners made two visits to the lab, separated by 7–15 days (*M* = 10.44 days, *SD* = 2.69), while the twenty-eighth participant could only return after 78 days. All stimulus presentation routines were programmed and run in MATLAB v7.1 (The Mathworks, Inc., Natick, MA).

- **Sentences and Words.** For each task (Simple Sentences, Low Predictability Sentences and Words, respectively), each session featured a set of 70 different items with 10 at each difficulty level. Within each task, one set of 70 items was labeled as Set A and another 70 distinct items as Set B. Fourteen participants received Set A items in Session 1, while the remainder received Set B items in Session 1. Within-session, a pseudorandomization routine ensured that the 70 items (i.e., their linguistic content) were completely randomized across the task, but that within each chronological block of 7 sentences there was an example from each difficulty level.
- **Consonants and Vowels.** The consonants and vowels were tested separately. Each of the tokens was repeated at all of the seven difficulty levels, and the whole list of items was fully randomized. Exposure to the difficulty levels was not chronologically constrained.

In each session, the tasks were administered in the order: BKB sentences, IEEE sentences, words, consonants, vowels. All test materials were presented over Sennheiser HD25-SP headphones in a quiet room, at a fixed volume setting using QuickMix (Version 1.06; Product Technology Partners, Cambridge, UK). The sentences and words tasks were open-set recognition tasks. Each stimulus was played once and the participant gave a typed report of the item content. Responses were self-timed. The listener was encouraged to type as much as possible from what they heard (and that partial answers were acceptable), but were also told that it was fine to leave a blank response bar if the item was completely unintelligible. The consonants and vowels tasks each adopted a 17-alternative forced-choice paradigm. The response choices were presented on a printed sheet which remained in view for the duration of the task. In these two tasks, listeners were encouraged not to leave any gaps, even when they were completely unsure of the answer.

### Analysis

For each participant, performance on the tasks was scored as the proportion of keywords/items correct at each distortion level. For the sentences, a scoring system was adopted in which deviations in tense and number agreement on nouns (i.e., if the participant reported “men” when the actual keyword was “man”) and verbs (i.e., if the participant reported “carries” or “carried” when the correct word was “carry”) were allowed. The reasoning behind this approach was to allow for errors that may have resulted from the participant's attempts to report a grammatically correct sentence for each item. For example, if the participant hears the first keyword in “the cup hangs on a hook” as “cups,” then he/she may choose to report “hang” as the second keyword, in order to maintain number agreement. For both the Sentences and Words, typographic errors that produced homophones of the target word e.g., “bare” and “bear” were permitted.

#### Psychometric performance curves

**Figure 1:**
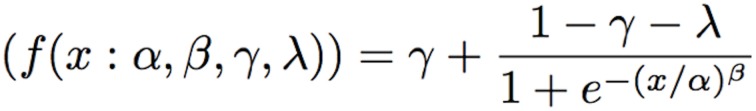
**Equation used to estimate psychometric functions describing the relationship between number of bands and speech intelligibility.** α, alpha; β, beta; γ, gamma; λ, lambda. “x” in this study was the log of the number of channels in the noise vocoder.

Logistic curve-fitting was performed on group data (by task and session), and on each individual data set (by participant, task, and session) using the psignifit software package (Wichmann and Hill, 2001a,b). For superior fits, the distortion levels (number of bands) were converted into their log_10_ equivalents (as used by Shannon et al., 2004). Data from undistorted stimuli were not included. The equation used for fitting is shown in Figure 1.

In the output of the fitting procedure, the alpha parameter corresponds to the number of bands giving 50% of maximum performance, and was extracted from each fitted curve for use in subsequent analyses. Lower alpha values indicate better performance. Beta is inversely proportional to the curve steepness. The parameter gamma corresponds to the base rate of performance (or “guessing rate”), while lambda reflects the “lapse rate” i.e., a lowering of the upper asymptote to allow for errors unrelated to the stimulus level. The software takes a constrained maximum-likelihood approach to fitting, where all four variables are free to vary, but where, in this case, gamma and lambda are constrained between 0.00 and 0.05. For the forced-choice tasks (Consonants and Vowels), the gamma parameter was set to 1/17.

#### Information transfer analyses

The forced-choice nature of the consonant and vowel tasks means that the data could be arranged into confusion matrices for use in an Information Transfer (IT) analysis (e.g., Miller and Nicely, 1955). IT analysis makes use of confusions (e.g., /b/ is mistaken for /d/) in speech identification tasks to measure the extent to which phonetic features (e.g., place of articulation, presence/absence of voicing) in the stimuli are transmitted accurately to the listener. The data are typically quantified in terms of the proportion or percentage of available bits of information in the stimuli that are accurately received by the listener. If no confusions are made in the participant's identification of a certain feature (e.g., vowel length), the IT score would be 1 or 100%, and correspondingly, if the participant's responses do not vary lawfully with the actual feature value, the score would be 0.

Unfortunately, as the participants' responses were made by typing the answers, rather than by selecting onscreen response options, some participants in the current experiment deviated from the forced-choice response constraints. This could take the form of omitted responses (which often occurred at particularly difficult distortion levels) or responses from outside the closed list. As a consequence, all data sets that included any omissions or deviations from the forced-choice options were not included in the IT analysis.

***Consonants.***A total of 14 data sets were entered into the IT analysis for consonant recognition. The feature matrix used included voicing, place and manner, and is shown in Table 1.

**Table 1:** **Feature matrix for IT analysis of the Consonants task**.

	**B**	**d**	**f**	**g**	**dƷ**	**k**	**l**	**m**	**n**	**p**	**s**	ʃ	**t**	**v**	**w**	**j**	**z**
Voicing	+	+	−	+	+	−	+	+	+	−	−	−	−	+	+	+	+
Manner	plos	plos	fric	plos	aff	plos	app	nas	nas	plos	fric	fric	plos	fric	app	app	fric
Place	bil	alv	lad	vel	paa	vel	alv	bil	alv	bil	alv	paa	alv	Lad	lav	pal	alv

For Voicing, the ‘+’ and ‘−’ signs correspond to present and absent voicing, respectively. For Manner, plos, plosive; fric, fricative; aff, affricate; app, approximant; nas, nasal. For Place, bil, bilabial; alv, alveolar; lad, labiodental; paa, postalveolar; vel, velar; lav, labialized velar; pal, palatal.

Unconditional IT feature analyses were run within the FIX analysis package (Feature Information XFer, University College London, UK; http://www.phon.ucl.ac.uk/resource/software.html). The amount of Information transferred for Voicing, Place, and Manner (as a proportion of the amount input for each of the features) was recorded for (i) group confusion matrices constructed at 1, 2, 4, 8, 16, and 32 bands, for Session 1 and Session 2 separately and (ii) for individual confusion matrices collapsed across 1–32 bands, for Session 1 and Session 2 separately. For the particular set of consonants used, there were 0.937 bits of information available for Voicing, 2.542 bits for Place of articulation and 2.095 bits for Manner of articulation.

***Vowels.***A total of 14 data sets were entered into the IT analysis for vowel recognition. The feature matrix used included vowel height, backness, roundedness, length, and whether the vowel was a monophthong or diphthong (Table 2).

**Table 2:** **Feature matrix for IT analysis of the Vowels task**.

	**æ**	**eɪ**	**ɑ** 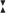	**ε** 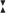	**i** 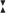	**ᴉə**	**e**	**ɪ**	**aɪ**	**ɜ**	**ɒ**	əʊ	**u:**	ɔ 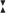	**aʊ**	ɔɪ	**Λ**
Height	no	cm-fc	o	om	c	nc-m	cm	nc	o-nc	om	o	m-nc	c	om	o-nc	om-nc	om
Backness	f	f-nf	b	f	f	nf-c	f	nf	f-nf	c	b	c-nb	b	b	f-nb	b-nf	b
Roundedness	n	n	n	n	n	n	n	n	n	n	y	ny	y	y	ny	yn	n
Length	s	l	l	l	l	l	s	s	l	l	s	l	l	l	l	l	s
Diphthong?	n	y	n	n	n	y	n	n	y	n	n	y	n	n	y	y	n

For Height, o, open; no, near-open; om, open-mid; m, mid; cm, close-mid; nc, near-close; c, close. For Backness, b, back; nb, near-back; c, central; nf, near-front; f, front. For Roundedness, y, rounded and n, unrounded. For Length, s, short and l, long. For Diphthong, y, diphthong and n, monophthong. Dashes indicate the separation of the diphthong descriptions into monophthongal elements, in temporal order.

IT feature analyses were run, using the FIX analysis package, for (i) group confusion matrices constructed at 1, 2, 4, 8, 16, and 32 bands, for Session 1 and Session 2 separately and (ii) for individual confusion matrices collapsed across 1–32 bands, for Session 1 and Session 2 separately. In all analyses, there were 3.264 bits of information available for vowel Height, 2.816 bits for Backness, 1.452 bits for Roundedness, 0.874 bits for Length and 0.937 bits for Mono—vs. Diphthong status.

All other reported statistical analyses were carried out in SPSS (version 19; IBM, Armonk, NY).

## Results

This section falls into two parts. In the first, psychometric performance functions are fitted to each individual's performance, and individual differences analyses of curve position used to characterize group performance across and within the two sessions. The second part uses IT analyses to explore the perception of consonants and vowels and relate this to recognition of sentences and words.

### Measuring profiles of learning and covariance

**Figure 2:**
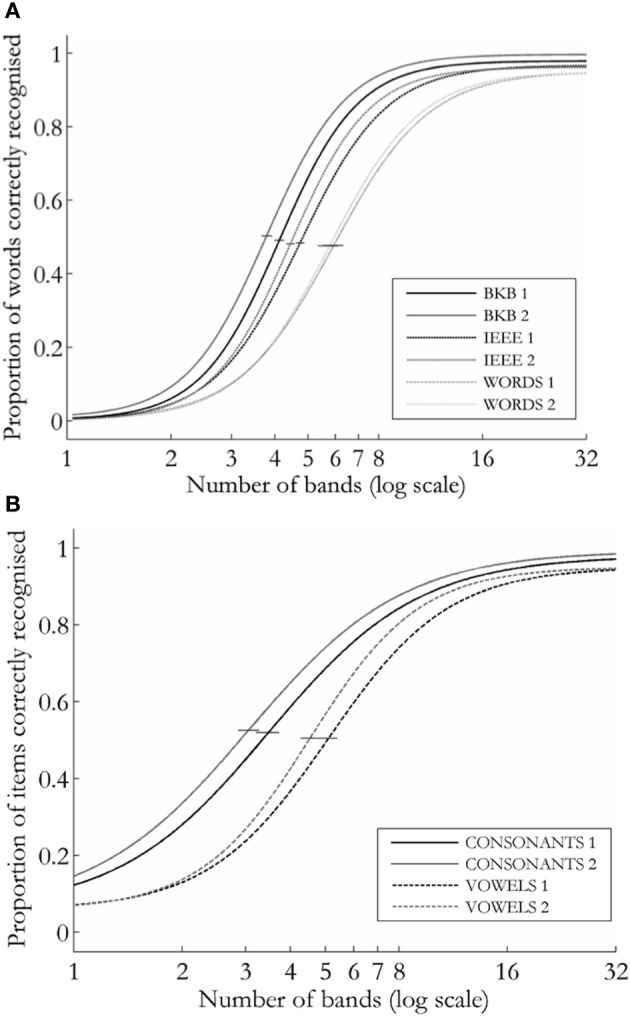
**Logistic curves describing group performance on the speech recognition tasks for (A) open-set tasks (sentences and words) and **(B)** consonants and vowels.** Error bars show 95% confidence limits around α.

Figure 2 shows a plot of the group performance functions for the open-set [2(a)] and closed-set [forced-choice; 2(b)] tasks in each session.

**Figure 3:**
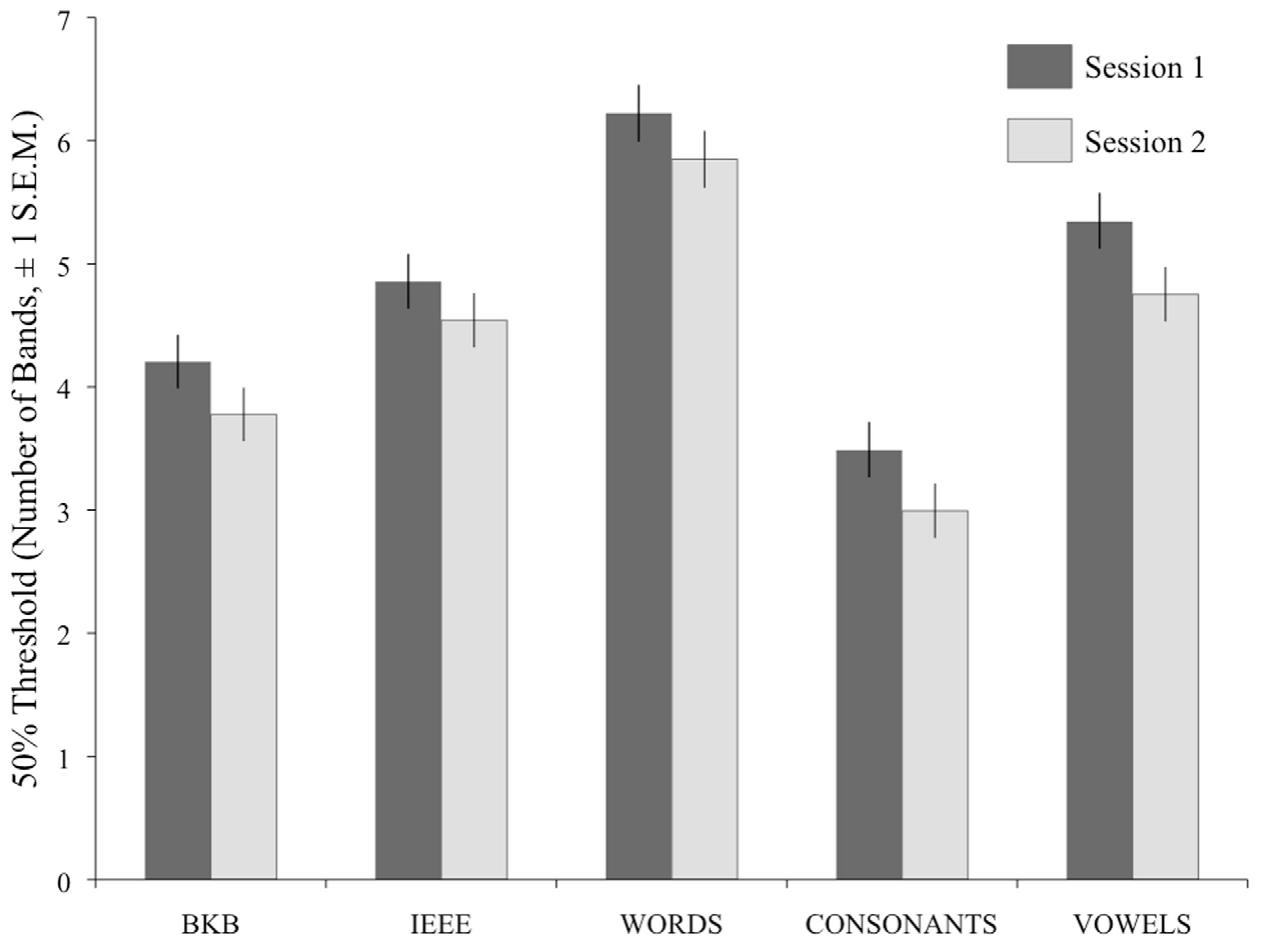
**Mean TNBs (Threshold Number of Bands) for speech recognition across the five tasks, and across the two test sessions.** Error bars show ±1 standard error of the mean.

For analysis, the alpha scores generated in the curve fitting procedure were operationalized as the Threshold Number of Bands (TNB) for each participant, in each task, in each session. Figure 3 shows that there was an overall decrease in TNBs on the five tasks between Session 1 and 2. A repeated-measures ANOVA analysis was run on the TNBs, with Session and Task as within-subjects factors. A between-subjects factor, Version (which coded the order of presentation of the item sets) was also included. There was a significant effect of Session [*F*_(1, 26)_ = 35.094, *p* < 0.001], a significant effect of Task [*F*_(4, 104)_ = 117.18, *p* < 0.001), and a non-significant interaction of these two factors (*F* < 1), indicating that the degree of improvement was not significantly different across tasks.

The between-subjects effect of Version was non-significant (*F* < 1), as were the two-way interactions of Version with Session [*F*_(1, 26)_ = 1.33, *p* = 0.260] and Version with Task [*F*_(1, 104)_ = 1.16, *p* = 0.333]. There was, however, a significant three-way interaction of Version, Session and Task [*F*_(4, 104)_ = 5.57, *p* < 0.001]; while most conditions across both versions showed a mean improvement from Session 1 to Session 2, Version A participants showed a trend in the opposite direction on the IEEE task, while the Version B participants showed a very small decrease in mean performance on the Words task from Session 1 to Session 2.

There was evidence of several significant relationships across tasks for the TNB scores. Table 3A shows the one-tailed Pearson's correlation matrix for TNB scores in Session 1. These show significant (and marginally significant) correlations between the two sentence tasks, and between the consonants and vowels tasks, while the words correlated reasonably well with all other tasks.

**Table 3:** **Pearson's correlation coefficients between the five tasks in the experiment, across the two testing session**.

	**BKB**	**IEEE**	**Words**	**Cons**	**Vowels**
**(A) SESSION 1**					
BKB	–	0.356^*^	0.259^∧^	0.003	−0.100
IEEE		-	0.323^*^	0.069	−0.056
Words			–	0.417^*^	0.331^*^
Cons				–	0.302^∧^
Vowels					–
**(B) SESSION 2**					
BKB	–	0.277^∧^	0.333^*^	0.299^∧^	0.236
IEEE		–	−0.025	0.393^*^	0.296^∧^
Words			–	0.015	0.057
Cons				–	0.317^∧^
Vowels					–

Cons, Consonants;

∧ p < 0.10,

* p < 0.05.

A common factor analysis was run on the threshold data, with maximum likelihood extraction and varimax rotation. The rotated factor matrix is shown in Table 4A, for those factors producing eigenvalues above 1. Two components were extracted. In the rotated matrix, the first component accounted for 22.6% of the variance, while the second component accounted for 19.2%. The pattern of correlations for TNB scores in Session 2 no longer fitted the processing framework suggested by the Session 1 data (see Table 3B), with the Words task now somewhat separate from the others. A common factor analysis was run on the data as for the Session 1 scores. This converged on two components—see Table 4B. In this analysis, Factor 1 accounted for 24.4% of the variance, where Factor 2 accounted for a further 20.4%.

**Table 4:** **Results of factor analyses on individual TNBs (Threshold Number of Bands)**.

	**Factor 1**	**Factor 2**
**(A) SESSION 1**		
BKB		0.605
IEEE		0.593
Words	0.705	0.469
Consonants	0.558	
Vowels	0.562	
**(B) SESSION 2**		
BKB	0.520	0.344
IEEE	0.545	
Words		0.946
Consonants	0.642	
Vowels	0.491	

Only factor loadings over 0.3 are shown.

### Exploring analytic listening using IT analysis

#### Consonants

**Figure 4:**
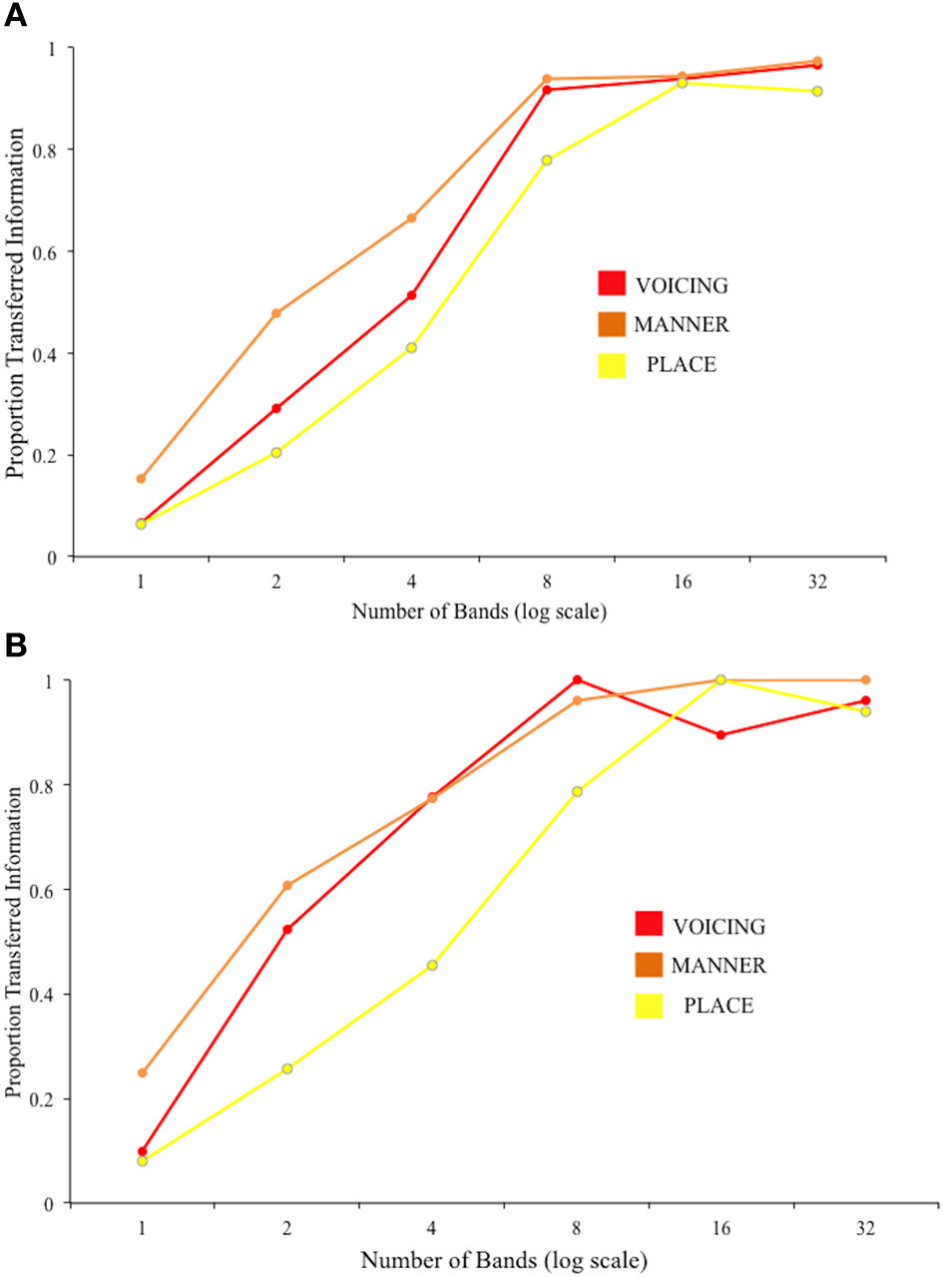
**Results of the group IT analysis on consonant perception for (A) Session 1 and (B) Session 2**.

The results of the pooled group analysis are plotted in Figure 4, showing the proportion of Information transferred for each feature, across each difficulty level. The plots give a readily interpretable visual representation of the “cue-trading” behavior of the listeners as spectral information is manipulated, and as a result of perceptual learning.

**Figure 5:**
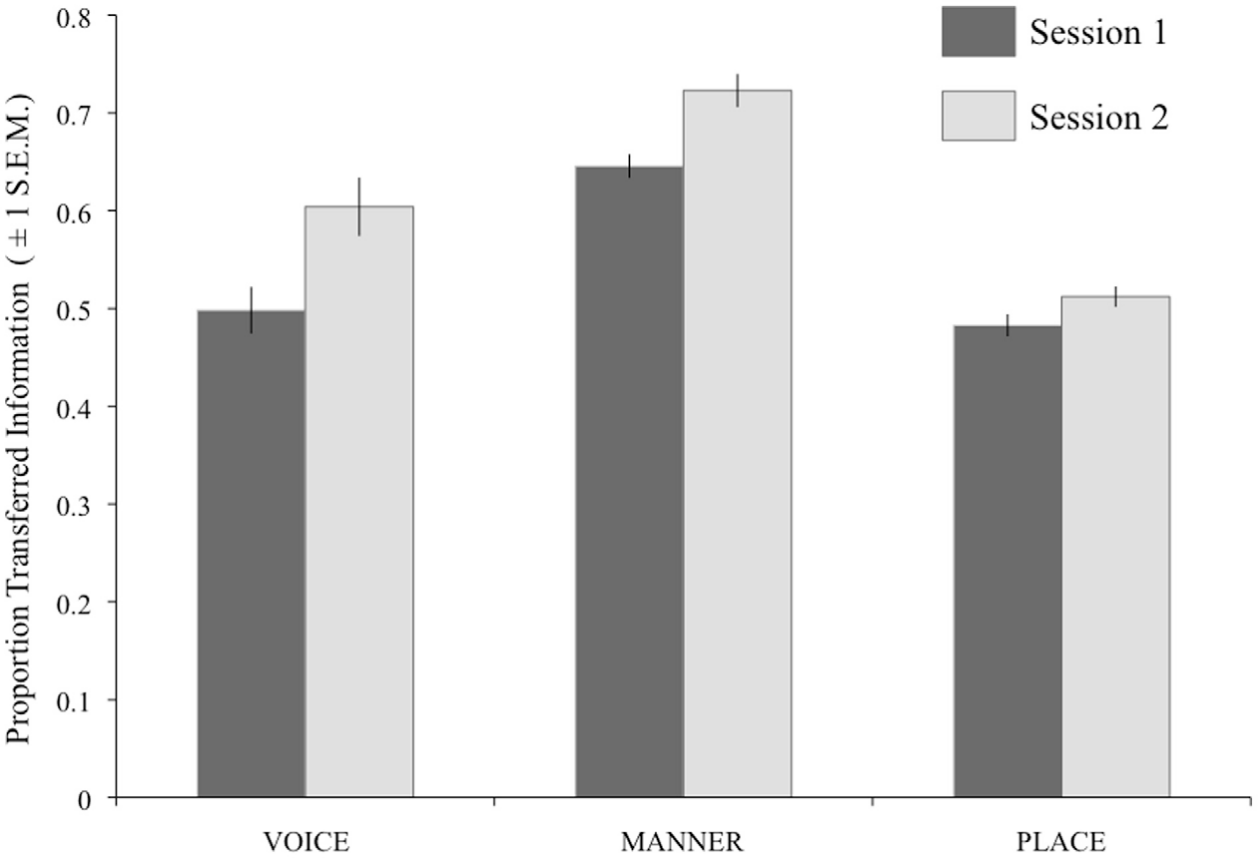
**Results of the IT analysis on consonant perception, using individual participant data.** For each feature, the darker bars show the results for Session 1, and the paler bars show the results for Session 2. Error bars show ±1 standard error of the mean.

Figure 5 shows the results of the individual analyses for each feature and session collapsed across difficulty level. A repeated-measures ANOVA gave significant effects of Session [*F*_(1, 13)_ = 13.52, *p* = 0.003] and Feature [*F*_(2, 26)_ = 64.13, *p* < 0.001]. A significant interaction of these two factors [*F*_(1.38, 26)_ = 4.16, *p* = 0.046; Greenhouse-Geisser corrected] was explored using 3 *post-hoc t*-tests with Bonferroni correction (significance level *p* < 0.017). These indicated a significant increase in IT for Manner [*t*_(13)_ = 2.97, *p* = 0.002] and Voicing [*t*_(13)_ = 2.97, *p* = 0.011] from Session 1 to Session 2, but not for Place [*t*_(13)_ = 2.17, *p* = 0.049].

The individual-subject IT scores for voicing, place and manner in each session were entered as predictors in linear regression analyses on the TNB scores for the five tasks. In Session 1, a significant model with Place and Voicing as predictors offered the best account of consonant recognition [*R*^2^adj. = 0.750; *F*_(2, 11)_ = 21.71, *p* = 0.001]. Performance on the vowels task was best predicted by Voicing [*R*^2^adj. = 0.295; *F*_(1, 12)_ = 6.44, *p* = 0.026]. In Session 2, Manner and Place predicted TNB scores on the Consonants task [*R*^2^adj. = 0.580; *F*_(1, 12)_ = 9.98, *p* = 0.003], while Manner scores predicted TNB scores on the IEEE sentences [*R*^2^adj. = 0.270; *F*_(1, 12)_ = 5.80, *p* = 0.033]. There were no other significant models.

#### Vowels

**Figure 6:**
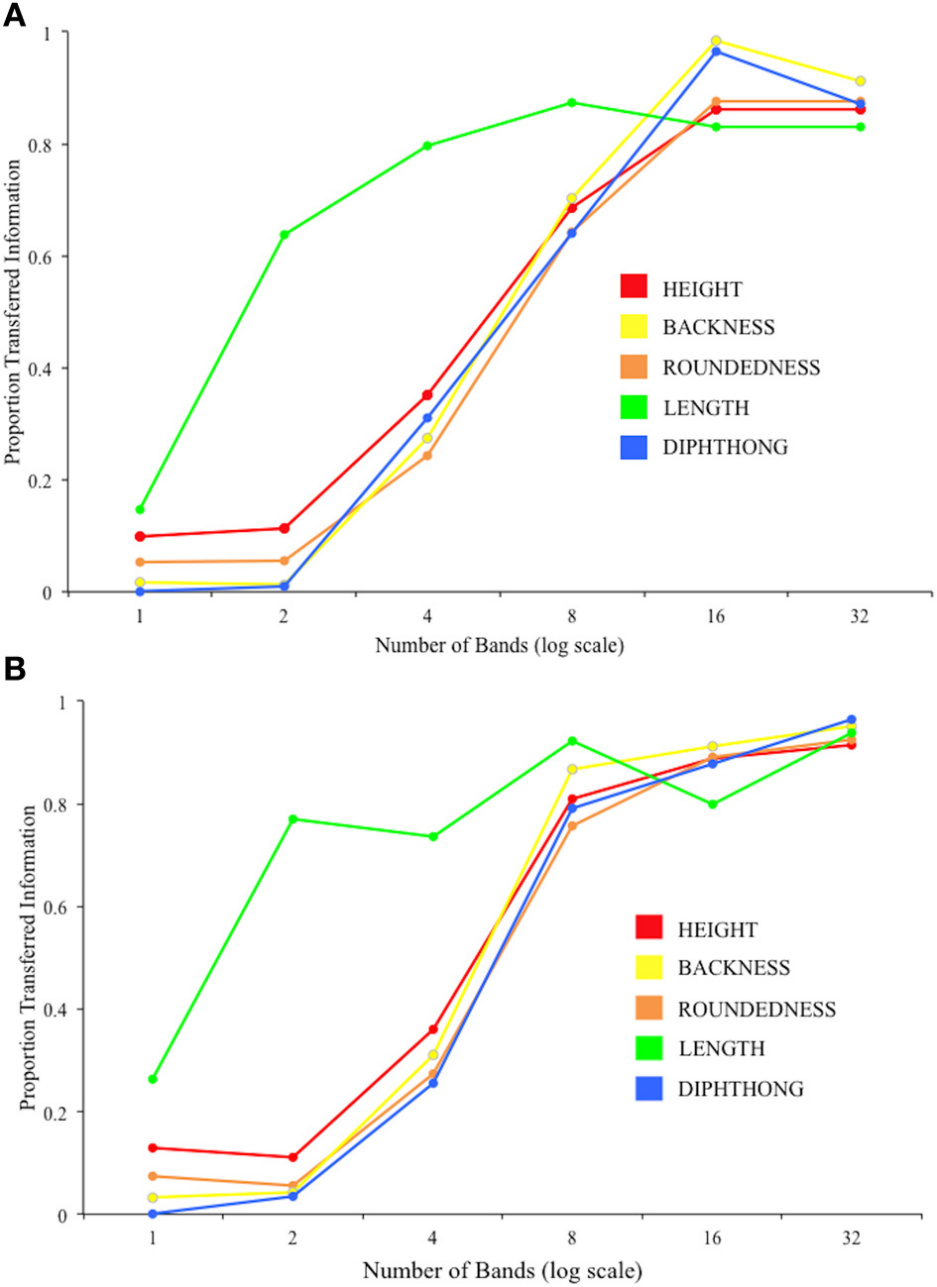
**Results of the group IT analysis on vowel perception for (A) Session 1 and (B) Session 2**.

The results of the pooled group IT (Figure 6) show that vowel Length information is the best transferred (as a proportion of the information input about this feature) of the five features at low spectral resolutions (1, 2, and 4 bands), with the other features more closely bunched. At greater spectral resolutions (16 and 32 bands), this discrepancy is reduced.

**Figure 7:**
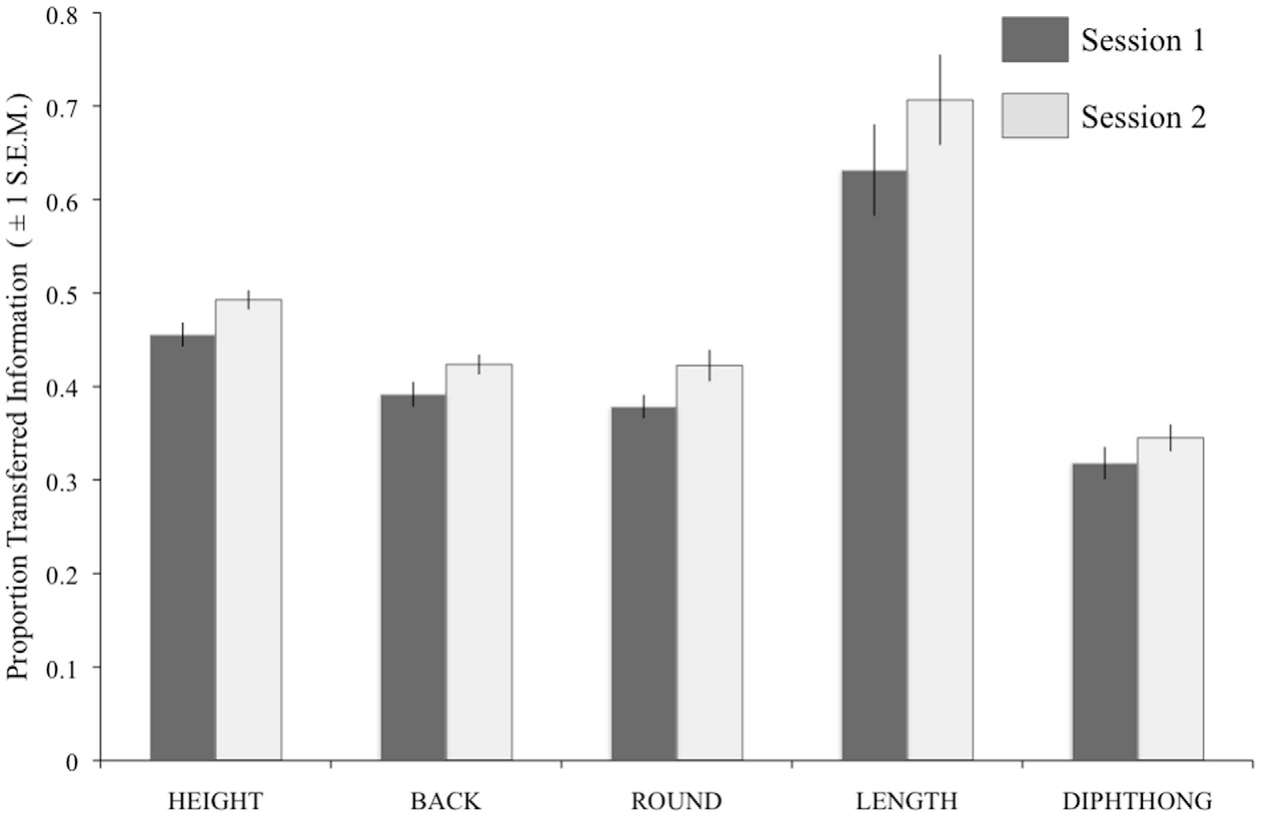
**Results of the IT analysis on vowel perception, using individual participant data.** For each feature, the darker bars show the results for Session 1, and the paler bars show the results for Session 2. Error bars show ±1 standard error of the mean. BACK, backness; ROUND, roundedness.

Figure 7 shows the results of the individual analyses for each Feature and Session collapsed across difficulty level). A repeated-measures ANOVA gave significant effects of Session [*F*_(1, 13)_ = 23.17, *p* < 0.001] and Feature [*F*_(1.12, 52)_ = 34.34, *p* < 0.001; Greenhouse-Geisser corrected) with no interaction of the two factors.

The individual-subject IT scores for each feature in each session were entered as predictors in linear regression analyses on the TNB scores for the five tasks. In Session 1, a significant model featured Height as the sole predictor of TNB scores on the Vowels task [*R*^2^adj. = 0.743; *F*_(1, 12)_ = 38.68, *p* < 0.001]. In Session 2, a significant model with Height and Length [*R*^2^adj. = 0.772; *F*_(1, 12)_ = 28.51, *p* < 0.001] gave the best prediction of TNB scores on the Vowels task, and a model with Length emerged as significant for scores on the BKB sentences [*R*^2^adj. = 0.308; *F*_(1, 12)_ = 6.80, *p* = 0.023]. There were no other significant models.

## Discussion

The current data showed evidence for improved recognition of noise-vocoded sentences, words and segments, when re-tested after a 1–2 week period of no exposure, and without any explicit training. Using individual differences as the starting point for analyses, we identified a pattern of covariance across levels of the linguistic hierarchy, which changed with learning. Analyses of confusion data revealed that participants in the experiment improved on the reception of acoustic-phonetic features by Session 2, but exhibited inefficient use of cues available in the vocoded signal. Further, these suggested predictive roles for specific phonetic features in the perception of noise-vocoded stimuli.

### Understanding noise-vocoded segments, words and sentences: effects of learning and task

We found that performance improved significantly between Session 1 and Session 2 of the experiment, and by an equivalent amount across tasks. As the experiment was primarily designed to explore individual differences and patterns of covariance across tasks and time, we chose to run the five speech tests in a fixed order for both sessions. Given that the task order was not counterbalanced across the group, we therefore cannot conclude whether the observed improvements are due to within-session exposure or between-session consolidation—for example, the BKB test occurred at the start of each session, and so the improvement observed by Session 2 may reflect adaptation during the remaining four tasks in Session 1 and before the delay period. However, taken across all tasks, the significant improvement in performance is, at least, a demonstration of medium-term retention of adaptation to noise-vocoded speech in the absence of exposure or training. We also note that, for the sentences and words tasks, the improved performance reflects perceptual learning at an acoustic-phonetic level, as recognition in Session 2 was tested using novel tokens (Davis et al., 2005).

### Understanding noise-vocoded segments, words and sentences: exploring covariance

Simple correlations between the tasks in each session showed, like Surprenant and Watson (2001), rather modest evidence for covariation of individual thresholds across the tasks (Table 3). This once again demonstrates that there is no straightforward, unitary approach to recognizing degraded speech across levels of the linguistic hierarchy. However, a factor analysis of Session 1 TNB scores suggested some systematicity—this revealed two similarly-weighted, orthogonal factors in the Session 1 threshold data, with sentences and words loading on one factor, and words and segments loading on the other. This suggests two independent modes of listening: a “top–down” mode making use of lexical, syntactic and semantic information to generate hypotheses about stimulus identity, and a “bottom–up” mode concerned with acoustic-phonetic discriminations. Notably, the words task occupies an intermediate status, by loading on both “top–down” and “bottom–up” factors. By Session 2, when performance had improved, all tasks but one—Words—patterned together. It appears that once the initial learning of sound-to-representation mappings has taken place, the listener can begin to approach most stimulus types in a similar way. In this sense, we suggest that the *nature* of the underlying factors was different in Session 2, such that these could no longer be well described by a greater involvement of “top–down” or “bottom–up” processes. However, we note that, in both session, the factors only accounted for a proportion of the variance, and therefore we cannot rule out the influence of additional factors underlying performance.

The plot in Figure 2 shows that the Words task was the most difficult of the open-set tasks in both sessions, with listeners requiring a greater amount of spectral detail (i.e., larger numbers of bands) in order to reach the 50% performance threshold. Within the open-set tasks, the overall amount of exposure to vocoded material across seventy sentences is much greater than for seventy monosyllabic words. However, Hervais-Adelman et al. (2008) showed that, even when matched for number of words of exposure, learning is still slower for noise-vocoded words than for sentences. These authors interpret such findings in terms of the relative richness of the “teaching signal” that assists learning. In the current experiment, the listener could draw upon many sources of knowledge against which to test hypotheses for sentence recognition—lexical, syntactic and semantic (Miller, 1947). Furthermore, the segment recognition tasks provided a learning frame-work through their forced-choice design. In contrast, the recognition of monosyllabic, degraded words could be constrained by the expectation of real lexical items, but with many monosyllables having several real-word neighbors (bat, cat, sat, fat etc.), any error in phonemic identification could lead to the participant making the wrong “guess” in their response to difficult items. In line with Hervais-Adelman et al., we argue that the nature of the Words task will have made it most difficult within each testing session, but also limited the potential for improved performance with learning. By the second testing session, listeners have established a sufficient level of acoustic-to-phonemic mapping that, in combination with expectancy constraints, allowed for improved performance on the sentences and segments tasks. However, the recognition of single words could not be performed using the same listening strategies(s).

### Exploring acoustic-phonetic processing: information transfer for consonants and vowels

The forced-choice design of the consonants and vowels tasks allowed us to explore performance in terms of the perception of phonetic features, using an IT analysis. The outcomes of IT analyses on the consonants and vowels recognition data are generally in agreement with the findings of several previous studies using noise-vocoded speech (Dorman et al., 1990, 1997; Shannon et al., 1995; Dorman and Loizou, 1998; Iverson et al., 2007). However, the current study enabled the assessment of two extra dimensions: the effect of perceptual learning on the extraction of feature information, and the relationship of feature processing to performance on the five speech recognition tasks.

The group IT analysis of the consonants data suggested that, numerically, place was the most poorly transferred feature, with no improvement across sessions. Dorman et al. (1990) tested identification of consonants by CI patients. They reasoned that, given the good temporal resolution by implants, envelope-borne information would be well transferred while the poor resolution offered by a small number of electrodes (6 in the device tested in their study) would limit the transfer of spectral information. Envelope information potentially cues listeners to voicing and manner, while transmission of place information is dependent on high-rate temporal structure (fluctuation rates from around 600 to 10 kHz) cueing spectro-temporal dynamics including the ability to resolve formants in the frequency domain; (Rosen, 1992). In CIs and their simulations, frequency resolution can be very poor in the region of formant frequencies, such that both F1 and F2 may be represented by the output of only one channel/electrode. Even if the first two formants can be resolved, the ability to differentiate one speech sound from the other can depend on within—formant transitions in frequency, for example in the discrimination of /b/ and /d/. The ability to make discriminations based on formant-carried frequency information in noise-vocoded speech will depend on the ability of listeners to compare the relative amplitude outputs of the different bands. A study by Shannon et al. (1995) with normal-hearing listeners exposed to noise-vocoded speech demonstrated that, after several hours of exposure, voicing and manner were almost completely transferred from spectral resolutions of 2 bands and upwards, while place IT was around 30% with 2 bands and did not exceed 70% by 4 bands. It should be noted that the three phonetic features of voicing, place and manner are not completely independent of each other, and there is likely to be some degree of overlap in the corresponding acoustic features. Dorman et al. (1990) point out that the amount of transferred place information should vary with the amount transferred about manner, as some manner cues facilitate place recognition e.g., frication manner (i.e., a wideband noise in the signal) potentially allows relatively easy discrimination between /s/ or /∫/ and /f/ or /θ/, as the former pair can be 15dB more intense than the latter.

The slightly more marked improvement in reception of voicing information than for the other two features in the current task can perhaps be explained by considering the acoustic nature of the noise-vocoded stimulus. Voicing can be weakly signaled by relatively slow envelope fluctations—for example, through detection of the longer silent periods in voiceless than voiced plosives, or in the greater amplitude of voiced compared to voiceless obstruents. However, voicing is also signaled by periodicity, that is, temporal regularity in the speech waveform carried by fluctuations primarily between 50 and 500 Hz (Rosen, 1992). This information is reasonably well preserved after the vocoding scheme used in the present experiment, where the amplitude envelope was low-pass filtered at 400 Hz. The between-session improvement shown for voicing at low band numbers could reflect the participants' increased ability to use the available temporal information in the stimulus to assist performance in the absence of cues to place of articulation that are more dependent on spectral resolution. However, voicing information is also carried by cues to overall spectral balance, as voicing is weighted toward low frequencies. These cues become apparent as soon as at least a second band of information is added to the noise-vocoded stimulus. We note that the duration of the preceding vowel in naturally-produced VCV stimuli can be a cue to voicing in the upcoming consonant. However, the mean preceding vowel duration was not significantly different between tokens with voiced and voiceless consonants in our task [*t*_(15)_ = 1.09, *p* = 0.292].

It is clear that the transmission of spectral shape information, as is required for identification of height, backness, roundedness, and diphthongs, is a limiting factor in recognition of noise-vocoded vowels, and that these four features are closely related in terms of recognition. However, as neither the amplitude envelope nor the duration of the signal is distorted by the noise-vocoding procedure, the information on vowel length should have been readily transmitted at all channels. Indeed, at lower numbers of bands, length was the most well recognized feature in the vowels task. However, overall recognition of this feature was well below 100%, and variable across the participant group. Regression models identified length as a significant predictor of scores on the other speech tasks, suggesting that timing and rhythmic information are of importance in perception of noise-vocoded speech. Our findings are similar to those by Iverson et al. (2007), who measured IT for vowel length in CI users and normal-hearing listeners listening to a CI simulation. Both listening groups in the Iverson et al. study showed sub-optimal IT. The authors propose that, given the excellent preservation of durational information in noise-vocoding, participants should be able to show 100% IT for length, even at low spectral resolutions. Therefore, while the evidence suggests that timing and rhythm may be important for successful perception of some forms of noise-vocoded speech, listeners may require more guidance and training in order to make better use of durational cues.

### Recognition of noise-vocoded speech over participant, task, and time—implications for training and cochlear implantation

The current study presents a number of findings relevant to more applied settings such as training regimes for CI recipients. We identified that, on initial exposure to noise-vocoded speech, the pattern of covariance across tasks was suggestive of two different “levels” of processing—one lexico-semantic (or “top–down”), and the other more acoustic-phonetic (or “bottom–up”). The words task was implicated in both of these factors. Isolated monosyllabic items such as “mice” and “gas” have lexical and semantic content, and the expectation of meaningful tokens can constrain the listener's candidate pool of targets in the recognition task. However, with all the items bearing the same CVC structure, and without any higher-order syntactic and semantic cues, the listener must also engage an analytical, acoustic-phonetic approach in order to successfully identify the words. It may be this demand on both approaches that makes the words task the most difficult in the set. Loebach and colleagues (Loebach and Pisoni, 2008; Loebach et al., 2008, 2009, 2010) argue that training in analytical, acoustic-phonetic listening offers the most promising route for adaptation to distorted speech. In a test of vocoded sentence perception they observed equivalent transfer of learning after training with semantically anomalous sentences as from training with real sentences. They suggest that this reflects an increased demand for attention to acoustic-phonetic aspects of the signal (rather than higher-order syntactic or semantic cues) when listening to the anomalous stimuli—their implication is that learning at this level can then be readily transferred to other stimulus types (Loebach et al., 2010). However, they also find that transfer is greatest between stimuli of the same linguistic class (Loebach and Pisoni, 2008). We have identified two sources of variability, of similar explanatory power, underlying the recognition of noise-vocoded speech. We suggest that both listening “strategies” should potentially yield benefits for adaptation, and that the effects observed by Loebach and colleagues may be associated with more generalized attentional engagement rather than the superior effects of analytic listening. Nogaki et al. (2007) partly ascribe the variability within normal-hearing participants listening to CI simulations to variable levels of enthusiasm and involvement in difficult listening tasks, and contrast this with the keener sense of urgency shown by CI patients, for whom successful training has important consequences for their quality of life. An experimental modulation of attentional engagement with noise-vocoded speech might offer greater insight into how this might differentially affect top–down and bottom–up aspects of listening. In the context of cochlear implantation, it is important to recognize that most of what we hear in everyday speech takes the form of connected phrases and sentences. Therefore, identifying methods of engaging attention in higher-order aspects of linguistic processing, such as the use of semantic and syntactic cues to “fill in the gaps” in difficult listening situations, may yield benefits of a similar magnitude to more bottom–up strategies.

We also identified targets for improved bottom–up processing of noise-vocoded speech from the current dataset. The use of IT analyses to explore acoustic-phonetic processing produced findings unattainable from basic recognition scores. We identified significant predictive roles for voicing and vowel length information in recognizing noise-vocoded stimuli across the linguistic hierarchy. Both of these properties were well represented at low spectral resolutions in the current stimuli—in particular, vowel length information was fully present even with one band. However, although perception of these features showed marked improvement over time, Figures 4–7 show that listeners' accuracy in recognizing these features was much less than 100% in both sessions. This suggests that, in the absence of specific guidance or instruction, listeners continue to rely on typically dominant cues to phoneme identification (e.g., formant frequencies in vowels) at the expense of other information that is more reliably preserved in the degraded signal. Based on this result, and similar findings from Iverson et al. (2007), we suggest that if CIs are to be trained in analytic listening to aid perceptual learning, this should be targeted at the acoustic cues that are most likely to be preserved when transmitted through the device. We suggest that focused training on perception of duration, amplitude modulation, and spectral balance cues could be used to improve acoustic-phonetic processing from the bottom–up by maximizing the usefulness of the information in the acoustic signal.

### Conclusion

The current study offers some insight into the existence of overlapping lexico-semantic and acoustic-phonetic processes underlying the adaptation to a CI simulation in normal-hearing participants. We suggest that both “top–down” and “bottom–up” listening strategies have potential validity in settings such as training for recipients of CIs. To improve “analytic” processing, we suggest that training should involve targeted attentional engagement with acoustic cues that are well preserved in the degraded stimulus. Further work is necessary to evaluate the benefits of such an approach. When considering all of the findings, however, we must acknowledge that the speech transformation used in the current study forms only a basic approximation to the signal perceived by most users of CIs. The process of implantation can result in incomplete insertion of the electrode array and damage to parts of the basilar membrane (yielding “dead regions”), both of which have consequences for the mapping of sound to the auditory nerve. Such effects can be simulated through additional transformations in the noise-vocoding technique (e.g., Rosen et al., 1999; Smith and Faulkner, 2006), and future work will need to determine whether the current findings are borne out for these more degraded signals.

### Conflict of interest statement

The authors declare that the research was conducted in the absence of any commercial or financial relationships that could be construed as a potential conflict of interest.
